# *Microhyla laterite *sp. nov., A New Species of *Microhyla* Tschudi, 1838 (Amphibia: Anura: Microhylidae) from a Laterite Rock Formation in South West India

**DOI:** 10.1371/journal.pone.0149727

**Published:** 2016-03-09

**Authors:** K. S. Seshadri, Ramit Singal, H. Priti, G. Ravikanth, M. K. Vidisha, S. Saurabh, M. Pratik, Kotambylu Vasudeva Gururaja

**Affiliations:** 1 Department of Biological Sciences, National University of Singapore, 14 Science Drive 4, Block S3, Singapore, Singapore; 2 Independent Researcher, B-14, Law Apartments, Karkardooma, Delhi, India; 3 Manipal University, Manipal, India; 4 Suri Sehgal Centre for Biodiversity and Conservation, Ashoka Trust for Research in Ecology and the Environment (ATREE), Royal Enclave, Sriramapura, Jakkur (P.O), Bengaluru, India; 5 Science Media Center, Gubbi Labs LLP, WS-5, I Floor, Entrepreneurship Center, Society for Innovation and Development, Indian Institute of Science Campus, Bengaluru, India; 6 Independent Researcher, A/103, Gokul, B. P. Road, Dahisar (W), Mumbai, India; 7 Independent Researcher, 3C/704, Whispering Palms, Lokhandwala Township, Kandivali East, Mumbai, India; University of Arkansas, UNITED STATES

## Abstract

In recent times, several new species of amphibians have been described from India. Many of these discoveries are from biodiversity hotspots or from within protected areas. We undertook amphibian surveys in human dominated landscapes outside of protected areas in south western region of India between years 2013–2015. We encountered a new species of *Microhyla* which is described here as *Microhyla laterite* sp. nov. It was delimited using molecular, morphometric and bioacoustics comparisons. *Microhyla laterite* sp. nov. appears to be restricted to areas of the West coast of India dominated by laterite rock formations. The laterite rock formations date as far back as the Cretaceous-Tertiary boundary and are considered to be wastelands in-spite of their intriguing geological history. We identify knowledge gaps in our understanding of the genus *Microhyla* from the Indian subcontinent and suggest ways to bridge them.

## Introduction

The Indian subcontinent supports a rich biological diversity despite high human population density [1–3]. This is attributed to the presence of a varied environment and habitats [1, 4], unique biogeographical and geological history [5] and a strong tradition of conservation of nature [1, 6]. The biodiversity across the country is not evenly distributed and is mainly concentrated in and around the Eastern Himalaya and the Western Ghats [4, 7]. Both these regions are known for a high level of endemism across taxa [8, 9].

The Indian subcontinent is one of few regions in the Old World to harbor a rich diversity of amphibians [10]. Currently, there are 391 species recorded from India, predominantly from the Western Ghats and Eastern Himalaya. A total of 220 amphibian species are recorded from the Western Ghats and several more are expected to be described in the near future. The species novelties being described are often from genera that are endemic to the Western Ghats (viz., *Beddomixalus*, *Ghatixalus*, *Indirana*, *Mercurana*, *Micrixalus*, *Nyctibatrachus*, *Raorchestes*) but such discoveries from widespread genera like *Microhyla*, *Hoplobatrachus*, *Fejervarya* and *Euphlyctis* are uncommon.

The genus *Microhyla* Tschudi, 1838 comprises of 38 extant species and is widespread across South and Southeast Asia [11]. There are currently eight valid species of Microhyla in India namely, *M*. *berdmorei* (Blyth 1856), *M*. *butleri* Boulenger, 1900, *M*. *chakrapanii* Pillai, 1977, *M*. *heymonsi* Vogt, 1911, *M*. *ornata* (Duméril and Bibron, 1841), *M*. *pulchra* (Hallowell, 1861), *M*. *rubra* (Jerdon, 1854) and *M*. *sholigari* Dutta and Ray, 2000 [11, 12].

We encountered a species of Microhyla during surveys between years 2013–2015 as part of a citizen science initiative, ‘My laterite: My habitat’ led by one of the authors (Ramit Singal). This species of *Microhyla* did not match descriptions of the eight known species from the region. We undertook further studies to determine its identity and here, we report: 1. the description of *Microhyla laterite* sp. nov., ascertained using molecular (12S and 16S rRNA genes), morphology and bioacoustic comparisons and 2. Assess the threat status of this species using IUCN Red List criteria.

## Materials and Methods

### Ethics Statement

Fieldwork and sampling was carried out in Manipal, Udupi District of Karnataka. All specimens were collected with permission from the Principal Chief Conservator of Forests and Chief Wildlife Warden, Karnataka State Forest Department (Permission No. PCCF(WL)/E2/CR-23/2015-16). Specimen collection and tissue sampling protocol followed guidelines for use of live amphibians and reptiles in field research by the American Society for Ichthyologists and Herpetologists and was approved by the Gubbi Labs Internal Committee on Animal Welfare and Ethics (Approval No. 2015-16/GLICAWE/01). All collections and tissue sampling adhered to the ethical standards put forth by the committee. Minimum samples were collected and used only for scientific work (*M*. *laterite* sp. nov: N = 4). Animals were located and gently picked up by hand and placed in moist cotton bags and transported to field station within 30 min. Individuals were then euthanized using 20% Benzocaine gel, a topical anesthetic. A small volume of the gel (< 0.5 cm^3^) was squeezed out on a swab and applied on the animals’ ventral region. After the animal ceased to show any signs of movement, a small portion of thigh muscle tissue (ranging between 0.1 to 0.25 cm^3^) was extracted for molecular analysis. A sterilized stainless steel scissor and forceps were used to incise tissue. Tissue was stored in molecular grade ethanol. Specimens were later fixed in 4% formalin for 24h and then transferred to 70% alcohol. Our animal handling protocols strictly following the guidelines for euthanasia of amphibians and use of Benzocaine 20% is well known to effectively minimize any pain or distress to the animal.

### Nomenclatural Acts

The electronic edition of this article conforms to the requirements of the amended International Code of Zoological Nomenclature, and hence the new names contained herein are available under that Code from the electronic edition of this article. This published work and the nomenclatural acts it contains have been registered in ZooBank, the online registration system for the ICZN. The ZooBank LSIDs (Life Science Identifiers) can be resolved and the associated information viewed through any standard web browser by appending the LSID to the prefix “http://zoobank.org/”. The LSID for this publication is: urn:lsid:zoobank.org:pub:BBBDC86C-FE35-4929-A484-62C25C497ACB. The electronic edition of this work was published in a journal with an ISSN, and has been archived and is available from the following digital repositories: PubMed Central, LOCKSS.

### Study Area

**Fig 1:**
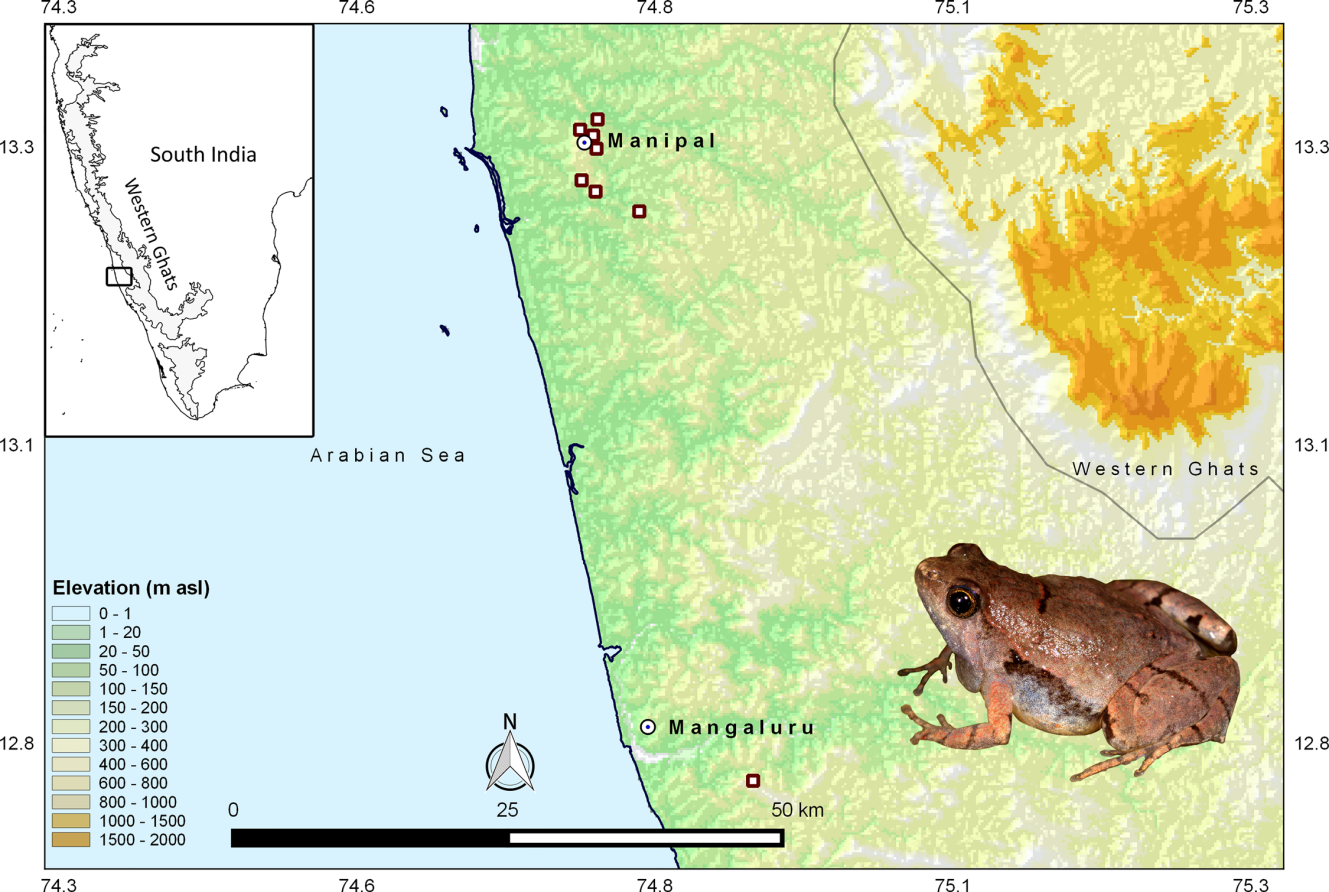
Map showing type locality of *M*. *laterite* sp. nov. White box with maroon outline: *M*. *laterite* sp. nov. Grey line indicates Western Ghats Boundary. Maps were generated using QGIS® Pisa Ver. 2.10. Data was sourced from www.gadm.org for administrative boundary of India and Shuttle Radar Topography Mission (SRTM) 90 m database (http://srtm.csi.cgiar.org) for elevation.

This study was conducted along the West coast of India (Fig 1). The new species of *Microhyla* was observed between years 2013–2015 in laterite habitats in and around the coastal town of Manipal, Udupi District, Karnataka State, India (13.2868°–13.3757° N and 74.7795°–74.8731° E, 50 m amsl). The region receives an annual rainfall of about 4000 mm. These laterite formations are a part of the ‘Deccan Traps’ flood plain and are believed to have originated sometime during the mid-Tertiary [13]. The overall habitat comprises of grasses, herbs, shrubs and stunted trees interspersed with agricultural fields and houses.

### Voucher Collection

Fieldwork and sampling was carried out in Manipal, Udupi District of Karnataka. All specimens were collected with permission from the Principal Chief Conservator of Forests and Chief Wildlife Warden, Karnataka State Forest Department (Permission No. PCCF(WL)/E2/CR-23/2015-16). Specimen collection and tissue sampling protocol followed guidelines for use of live amphibians and reptiles in field research by the American Society for Ichthyologists and Herpetologists and was approved by the Gubbi Labs Internal Committee on Animal Welfare and Ethics (Approval No. 2015-16/GLICAWE/01). All collections and tissue sampling adhered to the ethical standards put forth by the committee. Minimum samples were collected and used only for scientific work (*M*. *laterite* sp. nov: N = 4). Animals were located and gently picked up by hand and placed in moist cotton bags and transported to field station within 30 min. Individuals were then euthanized using 20% Benzocaine gel, a topical anesthetic. A small volume of the gel (< 0.5 cm^3^) was squeezed out on a swab and applied on the animals’ ventral region. After the animal ceased to show any signs of movement, a small portion of thigh muscle tissue (ranging between 0.1 to 0.25 cm^3^) was extracted for molecular analysis. A sterilized stainless steel scissor and forceps were used to incise tissue. Tissue was stored in molecular grade ethanol. Specimens were later fixed in 4% formalin for 24h and then transferred to 70% alcohol. Our animal handling protocols strictly following the guidelines for euthanasia of amphibians and use of Benzocaine 20% is well known to effectively minimize any pain or distress to the animal.

We photographed the individuals before and after they were euthanized. Four individuals (three males and one female) of the *M*. *laterite* sp. nov. were collected on 26^th^ June 2015 between 19:00–21:00 h by KSS and RS from Manipal.

### Molecular Analysis

We followed the protocol by Vences et al. [14] for DNA extraction from thigh muscle tissue. PCR amplification and sequencing of 16S and 12S rRNA genes was carried out following Gururaja et al. [15]. Amplified products were then sequenced at Chromous Biotech, Bangalore, India. The sequences were manually checked using Chromas lite 2.01 (http://www.technelysium.com.au/chromas_lite.html). Sequence alignment was carried out using the MAFFT algorithm [16] and manually corrected using MEGA® V5.10 [17]. Sequences are deposited in GenBank (Accession numbers: KT600663, 664–KT600670, 671). The dataset was 1357 base pairs in length. Combined sequence data of 12S and 16S rRNA of 23 species of *Microhyla* are used for phylogenetic tree construction with *Uperodon variegatus* as an outgroup (**S1 Table**).

Maximum likelihood (ML) algorithm and Bayesian inference methods were used for phylogenetic analysis. The ML analysis was executed in RaxML v1.3 [18] with GTR+I+G model selected as the best-fit nucleotide substitution model in jModel test [19] for 1000 bootstrap replicates. The Bayesian analysis was performed in MrBayes 3.2.4 [20]. The combined data set of 16S and 12S gene fragments were used for the analysis with GTR+I+G selected as best fit models in jModel test. The Markov chain Monte Carlo analysis for the dataset was run for 50 million generations and trees were sampled every 500 cycles. The convergence of the runs was analyzed by assessing the split frequency standard deviations (<0.001) and potential scale reduction factor (PSRF ~1.0). The first 10% of the sampled trees were discarded as burn-in and remaining samples were used to generate majority rule consensus tree. For estimating the genetic divergence, un-corrected pairwise genetic distance between the species was calculated in MEGA 5.10.

### Morphological Measurements

Individuals were measured using a Mitutoyo® digital caliper to the nearest 0.1 mm. Measurements and terminology follow Seshadri, Gururaja [21] and measured features are abbreviated and listed in **S1 Appendix**. Measurements of fingers and toes were made in ImageJ® from photographs taken using a Nikon® D90 Digital camera with a Nikkor® 105 mm micro lens. QGIS® Pisa Ver. 2.10 was used for illustrating hand and foot.

### Statistical Analysis

For bioacoustic comparison, a Mann-Whitney U test in R v.3.1.3 [22] was used to compare the means of call characteristics.

### Call Recordings and Analysis

Calls were recorded using Sennheiser K6® unidirectional microphone coupled with a Marantz PMD 660® solid state recorder. Vocalizations with relatively higher signal to noise ratio were chosen and analyzed using Audacity Ver.1.3 (Beta) and Raven Pro Ver.1.5. Eight calls from two individuals of *M*. *laterite* sp. nov., were used. Call terminology follows Kok and Kalamandeen [23]. Duration, dominant frequency and number of pulse of each call were analysed. Air temperature and relative humidity were recorded using a Kestrel^®^ 4500 pocket weather tracker.

### Maps and Geographic Range Estimation

QGIS® Pisa Ver. 2.10 was used to generate maps of range and distribution of *M*. *laterite* sp. nov. Data was sourced from www.gadm.org for administrative boundary and SRTM 90 m Database (http://srtm.csi.cgiar.org) for elevation. Area under minimum convex hull was computed on occurrence points of frogs to estimate extent of occurrence for IUCN assessment as per existing criteria [24].

## Results

### Molecular Identification of *Microhyla laterite* sp. nov.

**Fig 2:**
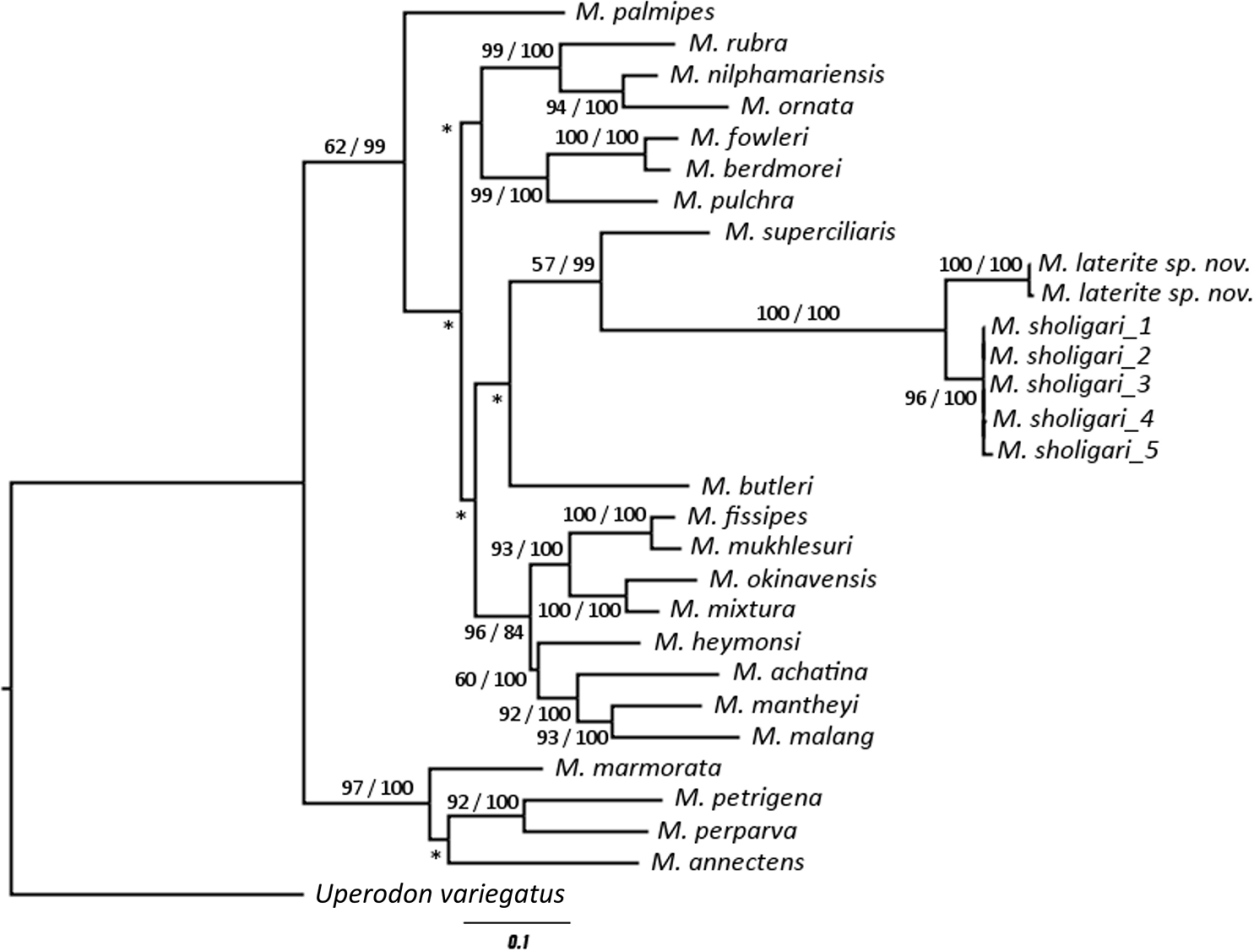
Maximum Likelihood tree for 23 *Microhyla* species and *Uperodon variegatus* as an outgroup. Numbers above and below the nodes indicate Bayesian Posterior Probabilities and Maximum Likelihood Bootstrap values >50, respectively. Asterisk indicates values <50.

The intra-specific un-corrected pairwise genetic distance (UPGD) of *M*. *laterite* sp. nov. was 0.0% (n = 2) and the inter-specific UPGD of *M*. *laterite* sp. nov. was lowest with *M*. *sholigari* (range: 4.8–5.03%, n = 5) and was most with *M*. *petrigena* (14.14%, n = 2). The UPGD between the 23 species of *Microhyla* used in the analysis varied from 4.8% to 14.14% (**S2 Table**). *Microhyla laterite* sp. nov. is a sister species of *M*. *sholigari*, forming a distinct clade as inferred from the phylogenetic tree (Fig 2). However, the relationships with other species are not fully resolved as indicated by the low bootstrap support. Therefore, *M*. *laterite* sp. nov. was described as a new species and compared with *M*. *sholigari*.

### Species Description

*Microhyla laterite* sp. nov.

urn:lsid:zoobank.org:act:321B8493-CD5C-4774-B664-6D6A36EAB0B2

Suggested common name: Laterite narrow-mouthed frog

Holotype: BNHS 5964, an adult male collected from laterite rocks in Kodanga, Herga village, Manipal, Udupi District by KSS and RS at 19:30–20:00 h on 26^th^ June 2015.

Paratypes: Two males (BNHS 5965, 5966) and one female (BNHS 5967) were collected in same locality, date and time as holotype by KSS and RS.

#### Diagnosis

The new species is assigned to the genus *Microhyla* owing to the following set of characters *sensu* Parker (25), Dutta and Ray (26) and Matsui, Hamidy [27]: Small sized adults with circular pupil; dorsal skin smooth, with markings from back of eye to vent; supratympanic fold present; paratoid glands absent; fingers without webbing, free with or without dilations; tongue oval, entire and free at the base; snout less than twice the diameter of eye; tympanum hidden by skin; palmar tubercles distinct; distinct oval shaped inner metatarsal tubercle and rounded outer metatarsal tubercle; rudimentary webbing in foot.

*Microhyla laterite* sp. nov. can be distinguished from all other congeners in the Indian subcontinent by the following suite of characters: (i) A very small sized adult frog (Male: 15.3–16.6 mm, n = 3 and Female: 18.4 mm, n = 1); (ii) snout obtuse in dorsal and ventral view with indistinct canthus rostralis, snout protrudes beyond mouth in ventral view (iii) tongue obovate, margin irregular, without lingual papilla (iv) tympanum hidden; (v) head wider than long; (vi) skin smooth on dorsum and venter; (vii) short, dark horizontal band on dorsum on the same plane as forelimbs. (viii) Throat with dense purplish-black pigmentation, reducing in intensity towards belly; (ix) reduced webbing in feet; (x) discs with circum-marginal groves on fingers and toes.

#### Description of holotype

**Fig 3:**
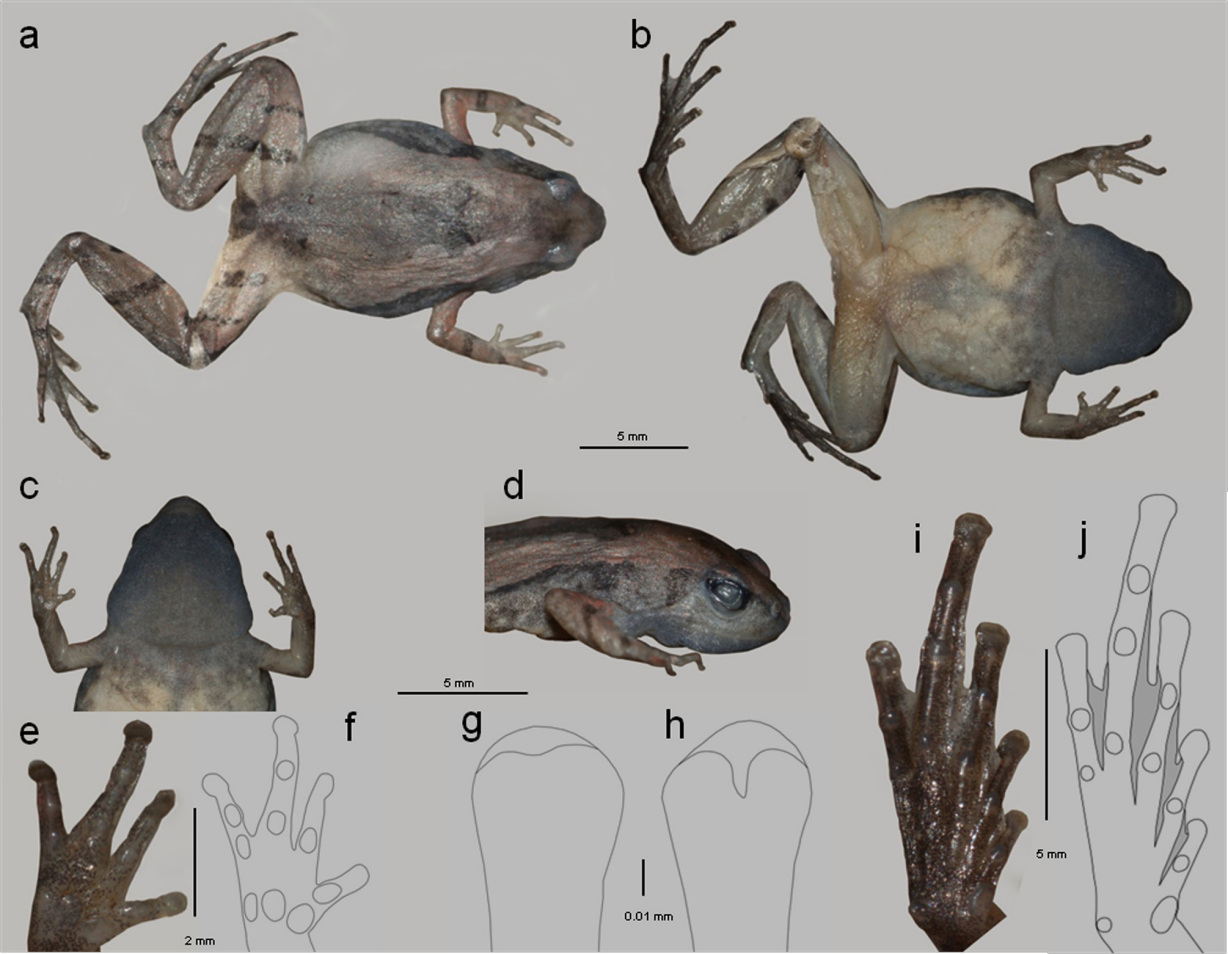
Plate depicting morphology of *M*. *laterite* sp. nov., adult male: BNHS 5964. (a) Dorsal view. (b) ventral view. (c) ventral view of throat depicting sub-gular skin fold and purplish pigmentation. (d) lateral profile showing tympanic region. (e) ventral view of hand. (f) line drawing of hand depicting palmar and sub-articular tubercles. (g) groove on third finger tip in male. (h) fourth toe tip showing distal notch in male. (i) ventral view of feet. (j) line drawing of feet depicting webbing, tarsal and sub-articular tubercles.

**Fig 4:**
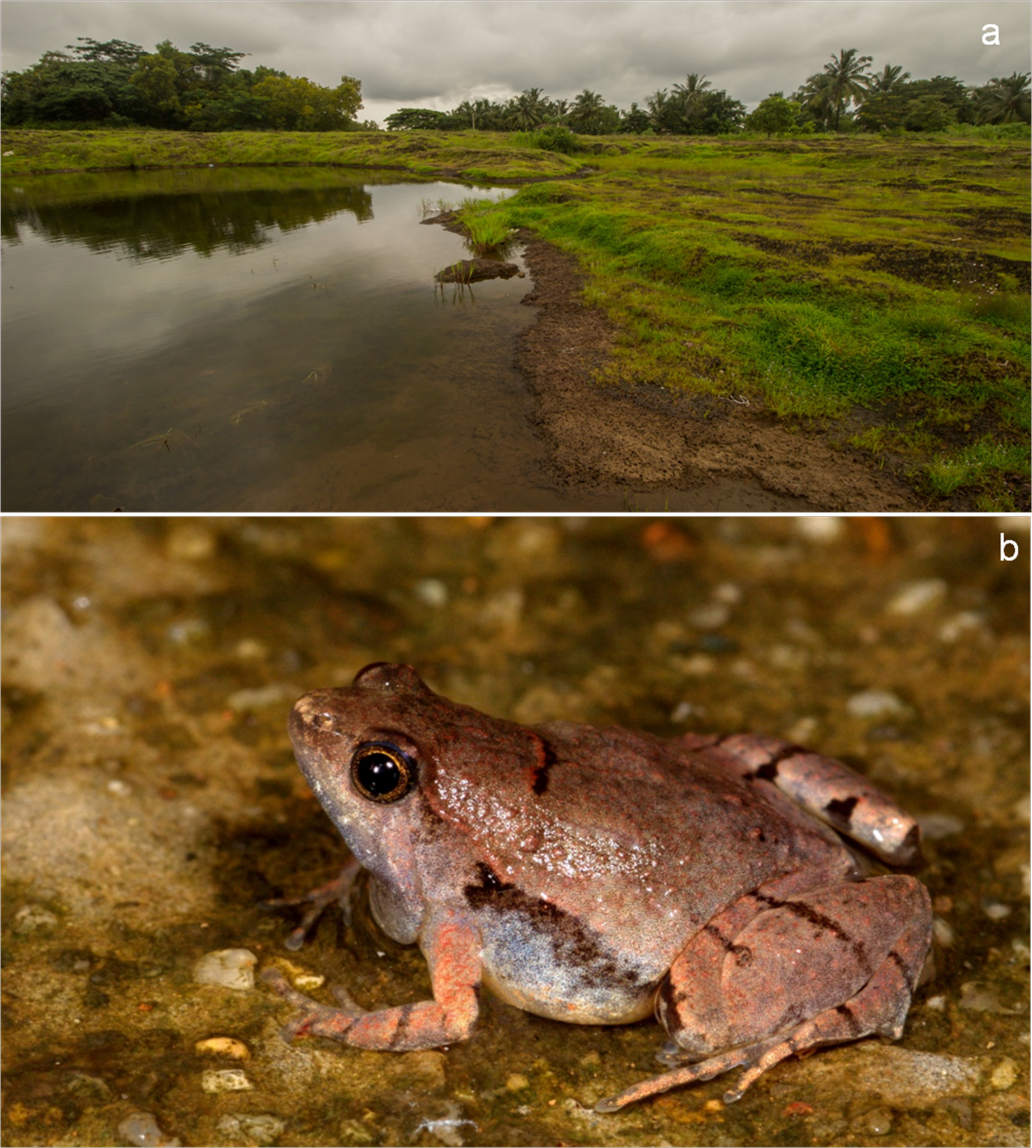
Plate depicting a laterite pool habitat of *M*. *laterite* sp. nov. and adult male in life (a) laterite pool habitat at type locality. (b) Dorso-lateral view of adult male.

A small sized adult (SVL = 16.6, male, BNHS 5964, all measurements in mm, Figs 3A–3J and 4A and 4B, Table 1.), head wider than long (HW = 4.4; HL = 3.9). Snout acute in both dorsal and ventral views, upper jaw protrudes slightly in ventral view. Snout acuminate in lateral profile. Snout 1.4 times longer the eye length (SL = 2.1; EL = 1.9). Canthus rostralis rounded. Loreal region concave. Interorbital space sloping towards snout, 1.2 times larger than upper eyelid width and sub-equal to internarial distance (IUE = 1.2; UEW = 1.0; IN = 1.2). Distance between posterior margins of eyes 1.5 times that of anterior margins (IBE = 3.6; IFE = 2.3). Nostrils rounded, without flap, closer to tip of snout than to eye (NS = 0.7; EN = 1.1). Symphysial knob present, weak. Tongue relatively large, obovate, free at base and with irregular margin. Lingual papilla absent. Vomerine teeth absent. Tympanum hidden, moderate supratympanic fold. Single sub-gular vocal sac with pair of openings at the base of lower jaw. A prominent sub-gular skin fold on throat. Eyes small (EL = 1.9), pupil-rounded.

**Table 1:** Morphometric measurements of *M*. *laterite* sp. nov. All measurements in mm. All individuals collected on 26^th^ June 2015 from Manipal. For abbreviations see S1 Appendix. Prefix BNHS to voucher numbers.

Type Voucher	Holotype 5964	Paratype 1 5965	Paratype 2 5966			Paratype 3 5967
Sex	M	M	M	Average ± SD	Range	F
**SVL**	16.6	15.3	15.9	15.92 ± 0.62	16.6–15.3	18.4
**HW**	4.4	4.6	5.2	4.74 ± 0.40	5.2–4.4	5.3
**HL**	3.9	3.8	4.2	3.96 ± 0.17	4.2–3.8	4.8
**HD**	3.3	4.2	3.2	3.57 ± 0.54	4.2–3.2	4.6
**IUE**	1.2	1.6	1.3	1.40 ± 0.20	1.6–1.2	1.8
**UEW**	1.0	1.2	1.1	1.10 ± 0.13	1.2–1.0	1.1
**SL**	2.1	2.0	2.1	2.07 ± 0.06	2.1–2.0	2.0
**EL**	1.9	2.0	1.8	1.86 ± 0.09	2.0–1.8	1.4
**MN**	3.1	3.4	3.3	3.25 ± 0.16	3.4–3.1	3.5
**MFE**	2.5	2.3	2.6	2.44 ± 0.13	2.6–2.3	2.6
**MBE**	1.7	1.7	1.8	1.74 ± 0.06	1.8–1.7	1.7
**IN**	1.2	1.4	1.6	1.36 ± 0.20	1.6–1.2	1.7
**IFE**	2.3	2.6	2.6	2.51 ± 0.16	2.6–2.3	2.6
**IBE**	3.6	4.2	4.4	4.07 ± 0.39	4.4–3.6	4.3
**NS**	0.7	0.8	0.9	0.80 ± 0.07	0.9–0.7	1.4
**EN**	1.1	1.3	1.1	1.14 ± 0.12	1.3–1.1	1.0
**FLL**	3.0	3.0	3.2	3.07 ± 0.11	3.2–3.0	3.2
**HAL**	3.9	3.9	3.8	3.90 ± 0.06	3.9–3.8	4.4
**FD1**	0.5	0.2	0.2	0.30 ± 0.14	0.5–0.2	0.3
**FD2**	0.4	0.3	0.2	0.30 ± 0.07	0.4–0.2	0.4
**FD3**	0.4	0.3	0.4	0.36 ± 0.09	0.4–0.3	0.4
**FD4**	0.5	0.4	0.4	0.41 ± 0.07	0.5–0.4	0.5
**FW1**	0.5	0.3	0.2	0.31 ± 0.14	0.5–0.2	0.3
**FW2**	0.4	0.3	0.2	0.28 ± 0.08	0.4–0.2	0.3
**FW3**	0.3	0.3	0.3	0.32 ± 0.03	0.3–0.3	0.3
**FW4**	0.4	0.4	0.3	0.36 ± 0.02	0.4–0.3	0.4
**FIL**	1.3	0.6	0.8	0.89 ± 0.39	1.3–0.6	1.1
**FIIL**	1.9	1.2	1.0	1.37 ± 0.51	1.9–1.0	1.2
**FIIIL**	2.3	2.3	2.0	2.20 ± 0.17	2.3–2.0	2.2
**FIVL**	2.0	1.7	1.2	1.61 ± 0.42	2.0–1.2	1.6
**FL**	7.0	7.9	7.6	7.48 ± 0.46	7.9–7.0	8.2
**ShL**	8.3	7.5	8.2	8.01 ± 0.42	8.3–7.5	10.4
**TW**	1.9	2.3	2.3	2.13 ± 0.24	2.3–1.9	2.6
**FOL**	8.0	8.3	7.9	8.07 ± 0.21	8.3–7.9	9.9
**TW1**	0.3	0.3	0.3	0.31 ± 0.03	0.3–0.3	0.3
**TW2**	0.3	0.5	0.3	0.37 ± 0.09	0.5–0.3	0.4
**TW3**	0.4	0.5	0.4	0.43 ± 0.07	0.5–0.4	0.4
**TW4**	0.4	0.4	0.3	0.40 ± 0.05	0.4–0.3	0.3
**TW5**	0.4	0.4	0.3	0.39 ± 0.05	0.4–0.3	0.3
**TD1**	0.3	0.4	0.3	0.34 ± 0.06	0.4–0.3	0.3
**TD2**	0.4	0.5	0.4	0.42 ± 0.08	0.5–0.4	0.5
**TD3**	0.5	0.6	0.5	0.51 ± 0.06	0.6–0.5	0.5
**TD4**	0.6	0.5	0.5	0.53 ± 0.03	0.6–0.5	0.4
**TD5**	0.5	0.5	0.4	0.46 ± 0.05	0.5–0.4	0.3
**IMT**	0.6	0.5	0.7	0.60 ± 0.12	0.7–0.5	0.8
**OMT**	0.3	0.4	0.5	0.38 ± 0.08	0.5–0.3	0.4
**TFOL**	12.2	11.6	10.8	11.54 ± 0.70	12.2–10.8	14.7
**TIL**	1.0	1.1	1.1	1.05 ± 0.03	1.1–1.0	1.1
**TIIL**	1.7	1.7	1.3	1.53 ± 0.22	1.7–1.3	1.7
**TIIIL**	3.0	3.2	2.2	2.79 ± 0.50	3.2–2.2	3.3
**TIVL**	4.6	5.0	4.2	4.63 ± 0.38	5.0–4.2	5.1
**TVL**	2.7	3.0	2.6	2.74 ± 0.22	3.0–2.6	3.0
**MTFF**	4.8	4.1	4.2	4.37 ± 0.36	4.8–4.1	5.3
**MTTF**	4.9	4.4	4.7	4.64 ± 0.23	4.9–4.4	5.0
**TFTF**	3.7	3.1	3.0	3.26 ± 0.41	3.7–3.0	4.1
**FFTF**	3.7	3.4	3.3	3.48 ± 0.21	3.7–3.3	5.1

Fore limb shorter in length than hand (FLL = 3.0; HAL = 3.9). Dermal fringe present on fingers. Webbing between fingers absent. Relative lengths of fingers I<II<III≤IV (FL I = 1.3; FL II = 1.9; FL III = 2.3; FL IV = 2.0). Finger tips with disc (FD I = 0.5, FD II = 0.4, FD III = 0.4, FD IV = 0.5; FW I = 0.5, FW II = 0.4, FW III = 0.3, FW IV = 0.4). Circum-marginal groove present and notched distally. Palmar tubercles well developed and distinct. Outer tubercle divided in two. Subarticular tubercles distinct (finger: i = 1, ii = 1, iii = 2, iv = 2) rounded. Supernumerary tubercles present. Nuptial pad absent. Hind limbs moderately long, touch when folded at right angles to body. Shank 4.4 times longer than wide (ShL = 8.3; TW = 1.9), longer than thigh length (TL = 7.0) and longer than foot length (FOL = 8.0). Heel to tip of fourth toe (TFOL = 12.2) about 2.88 times longer than fourth toe length (ToL IV = 4.6). Relative toe length I<II<III<V<IV (ToL I = 1.0; ToL II = 1.7; ToL III = 3.0; ToL IV = 4.6; ToL V = 2.7). Toe tips dilated (TD I = 0.3, TD II = 0.4, TD III = 0.5, TD IV = 0.6, TD V = 0.5; ToW I = 0.3, ToW II = 0.3, ToW III = 0.4, ToW IV = 0.4, ToW V = 0.4). Webbing reduced (MTTF = 4.9, MTFF = 4.8, TFTF = 3.7, FFTF = 3.7). Inner and outer metatarsal tubercle distinct. Inner metatarsal tubercle elongated (IMT = 0.6) larger than the rounded outer metatarsal tubercle (OMT = 0.3). Supernumerary tubercles and tarsal tubercle present (toe: i = 1, ii = 1, iii = 2, iv = 3-3^rd^ weak, v = 2-2^nd^ weak).

#### Skin texture in preservative

Skin on snout, inter-orbital space and sides of head smooth; Dorsum smooth, interspersed with tubercles increasing in intensity towards vent; Dorsal surface of forelimb and hind limb smooth with tubercles on upper arm, thigh, shank and foot. Skin on ventral side smooth, throat shagreened.

#### Color in preservative

Dorsal coloration pale brown; tubercles pale red; a short dark horizontal band on dorsum along the same plane as forelimbs; a pair of black spots at the urostyle bone projections; forelimbs reddish brown with black cross bands. Tympanic region grayish black. Flanks with a black band starting above shoulder and terminating just before the groin. Anterior part of thigh with distinct black band starting from knee and terminating short of groin. Dorsal surface of hind limbs brownish with black cross bands. Vent with a black triangular marking. Anterior and posterior portions of pupil black. Ventral region pale cream colored; throat with dense purplish-black pigmentation, reducing in intensity towards belly; tarsus to tip of toes brownish with pale buff colored webbing.

#### Color in life

Overall pale brown with prominent black markings on dorsum, hands, feet and flanks. Distinct black horizontal band on dorsum along the same plane as forelimbs. The band has a red leading edge. Deep purplish black vocal sac when calling. Iris golden yellow with brown mottling. Pupil black. Ventral parts cream white except throat (Fig 4B).

#### Variations

Sexes dimorphic, female larger than male (SVL: male, 15.3–16.6 mm, female: 18.4 mm); Sub-gular skin fold absent and when gravid, un-pigmented eggs visible near flanks, belly and groin.

#### Etymology

This species is named after the laterite rock formations in the type locality and other parts of its geographic range (Fig 4A). The specific name is an invariable noun in the nominative singular in apposition to generic name.

### Comparisons

Comparisons were based on publications [25, 26, 28–30]. Morphologically, *M*. *laterite* sp. nov. is distinct from the sister species *M*. *sholigari* in the following set of characters (*M*. *laterite* sp. nov. vs. *M*. *sholigari*, n = 3 males, all values are ratios to SVL): (i) SVL/IUE larger (11.34 vs. 8.13), (ii) SVL/MBE smaller (9.14 vs. 12.44), (iii) SVL/NS larger (19.98 vs. 15.56), (iv) SVL/EN larger (14.00 vs. 12.88), (v) SVL/TW larger (7.46 vs. 6.39), (vi) SVL/IMT smaller (41.78 vs. 43.18), (vii) SVL/TFTF larger (4.87 vs. 3.29), (viii) SVL/FFTF larger (4.57 vs. 3.21). Additionally, *M*. *laterite* sp. nov. can be distinguished from *M*. *sholigari* by the presence of purplish black vocal sac vs. vocal sac cream with sparse brown pigmentation; dorsal pattern less constricted vs. dorsal pattern strongly constricted; webbing in feet reaching distal tubercle on fourth toe on the inside vs. webbing reaching proximal tubercle on fourth toe on the inside.

*Microhyla laterite* sp. nov. is also compared with four other species occurring in southern India and Sri Lanka and can be distinguished easily based on the characters below: *Microhyla ornata* and *M*. *rubra* are distinct from *M*. *laterite* sp. nov. as they lack finger and toe discs. *Microhyla zeylanica* is distinct from *M*. *laterite* sp. nov. in having a bluntly rounded snout vs. obtuse; elongate ridges and circular warts on dorsum vs. absent and digital discs without a cleft vs. cleft present. *Microhyla karunarathnei* differs from *M*. *laterite* sp. nov. in having toes with dermal fringe vs. absent; venter white with black marbling vs. venter crème white without marbling and vocal sack black with white stippling vs. vocal sac dark purplish black without stippling.

### Ecology and Natural History Observations

*Microhyla laterite* sp. nov. was observed in and around the type locality during the south west monsoon seasons between April–August from 2013–2015. The species was found to be restricted to laterite habitats near rural and peri-urban areas. This species inhabits ephemeral ponds and other marshy areas in laterite habitats. They also occur in wet paddy fields where they were observed to vocalize from the embankment. Vocalization begins about 18:00 h and peaks between 19:15–21:30 h. The vocalization is very similar to that of ground crickets. Males can be located on leaf litter and other debris in dense grass clusters and often change their position while vocalizing. Upon disturbance, a few males were observed to stop calling and retreat backwards into small cavities in laterite rock formations where they lay low and hide for a few minutes.

The tadpoles of *M*. *laterite* sp. nov. are small, blackish overall and seen in shoals of over 100 individuals. We have observed *Euphlyctis mudigere* Joshy, Alam, Kurabayashi, Sumida, and Kuramoto, 2009, to feed on these tadpoles. Other species observed in the type locality are *M*. *ornata*; *Fejervarya sahyadris* (Dubois, Ohler, and Biju, 2001); *F*. *caperata* Kuramoto, Joshy, Kurabayashi, and Sumida, 2008; *Hoplobatrachus tigerinus* (Daudin, 1802); *Polypedates maculatus* (Gray, 1830) and *Euphlyctis cyanophlyctis* (Schneider, 1799).

### Geographic Range and IUCN Status

*Microhyla laterite* sp. nov. was found from very few locations in and around Manipal, Udupi District (between 13.2868°–13.3757° N and 74.7795°–74.8731° E, 50 m amsl) and Konaje, Mangaluru District (12.8183° N, 74.9319° E, 80m amsl) M.lateritii, Karnataka State (Fig 1). The geographic extent of occurrence was 146.13 km^2^. As per IUCN Red List criteria [24], this species qualifies to be listed as endangered (EN) under B1ab(iii),(iv).

### Bioacoustic Analysis and Comparison

**Fig 5:**
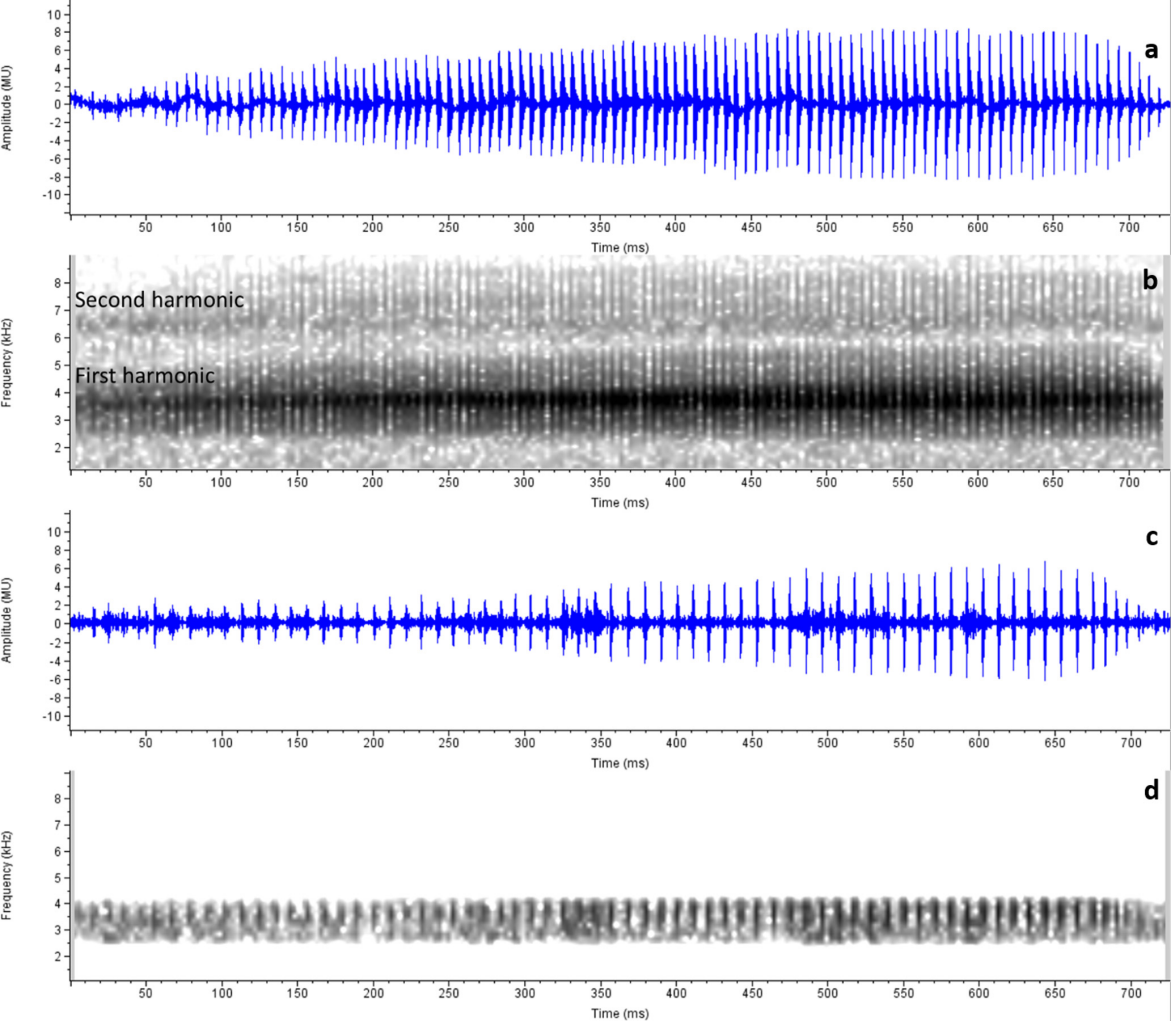
Amplitude and Spectrogram of advertisement calls. (a) Advertisement call of *M*. *laterite* sp. nov. A single call of 0.754s duration having 103 pulses, (b) a dominant frequency of 3664 Hz and 2^nd^ harmonics of 6506 Hz. (c) Advertisement call of *M*. *sholigari*. A single call of 0.726 s duration having 70 pulses and (d) a dominant frequency of 3518 Hz.

Calls of *M*. *laterite* sp. nov. were recorded on 26^th^ May 2015 between 17:30–18:30 h; Air Temperature: 27.9° C; Relative Humidity: 95% at Manipal 13.3593° N, 74.7979° E, 50 m amsl. The call sounds like ‘Zeeeeee….Zeeeee….Zeeeee…’ Calls of *M*. *laterite* sp. nov. (Fig 5A) had 90–126 pulses in each call (Mean ± SE, 106.88 ± 4.94, n = 8). Average dominant frequency was 3582.63 ± 15.70 Hz (Fig 5B; range: 3538–3664 Hz) and call duration was 0.72 ± 0.04 s (range: 0.60–0.85 s). We observed 2^nd^ harmonics in *M*. *laterite* sp. nov. at 6458 ± 35.64 Hz (range: 6326–6607 Hz). A sample audio of *M*. *laterite sp*. *nov*. is given as **S1 Audio**.

Calls of *M*. *sholigari* were recorded on 4^th^ July 2015, between 23:00 h and 23:45 h at Bisle, 12.7192° N and 75.6915° E, 837 m amsl. Air temperature was 23.25 ± 0.27° C and relative humidity of 92 ± 2%. Calls of *M*. *sholigari* had 64–72 pulses (Mean ± SE, 69.63 ± 1.18, n = 8) in each call (Fig 5C). Average dominant frequency was 3620.38 ± 26.41 Hz (Fig 5D; range: 3518–3779) and call duration was 0.72 ± 0.03 s (range: 0.53–0.81 s). A sample audio of *M*. *sholigari* is given as **S2 Audio**. Call duration and dominant frequency were not statistically significant between the two species (Mann Whitney U test: W = 33, P = 0.96 and W = 22.5, P = 0.34 respectively) however, the number of pulses were significantly higher in *M*. *laterite* sp. nov. (W = 64, P = 0.0009).

## Discussion

Members of the family Microhylidae comprise about 8% of all amphibians [31]. Having a pan tropical distribution, they have been of considerable interest in taxonomy and systematics. The classification and understanding of their evolutionary relationships has been in flux since Parker’s comprehensive monograph in 1934. Several studies have been undertaken but have resulted in contradictory findings [32, 33]. Further, identifying species in this genus based solely on morphology has proven to be difficult, owing to the small size, high morphological similarity and general diminutive habits of *Microhyla*. Hence, most species discoveries in South and Southeast Asia rely on integrative approach using morphology, molecular and bioacoustic data [34]. Recent studies on *Microhyla* from Indian subcontinent report several cryptic and un-described lineages [28, 31]. Currently, there are three species of *Microhyla* found in south India viz., *M*. *ornata*, *M*. *rubra* and *M*. *sholigari* in addition to *M*. *laterite* which is described here.

*Microhyla laterite* is named after the ‘lateritie’ or ‘ferricrete duricrust’ habitats where they commonly occur. Laterite formations are a common feature along the western slopes of the Western Ghats and West coast where they are native habitats and not degraded lands [13, 35, 36]. Laterite rocks are anisotropic in nature giving a heterogeneous composition of ferruginous compounds that form a rigid skeletal framework which is often impregnated with relatively softer clay; Cavities and pores are a characteristic structural irregularity of these rocks [36]. Recent evidence suggests that these rock formations formed between late Cretaceous and early Tertiary and have a complex geological history and are of paleontological importance [37].

These laterite plateaus are broadly considered as ‘rocky areas’ as they are usually devoid of trees and other woody vegetation and are therefore classified as wastelands [38]. Though devoid of tertiary vegetation, these areas are rich in microhabitats as there is an abundance of ephemeral pools and associated vegetation. Vegetation assessments have revealed laterite habitats to harbor a high diversity of plants, dominated by *Poaceae* with over 45 endemics [39]. *Utricularia* spp. and *Drosera* spp. are also common in these habitats.

**Fig 6:**
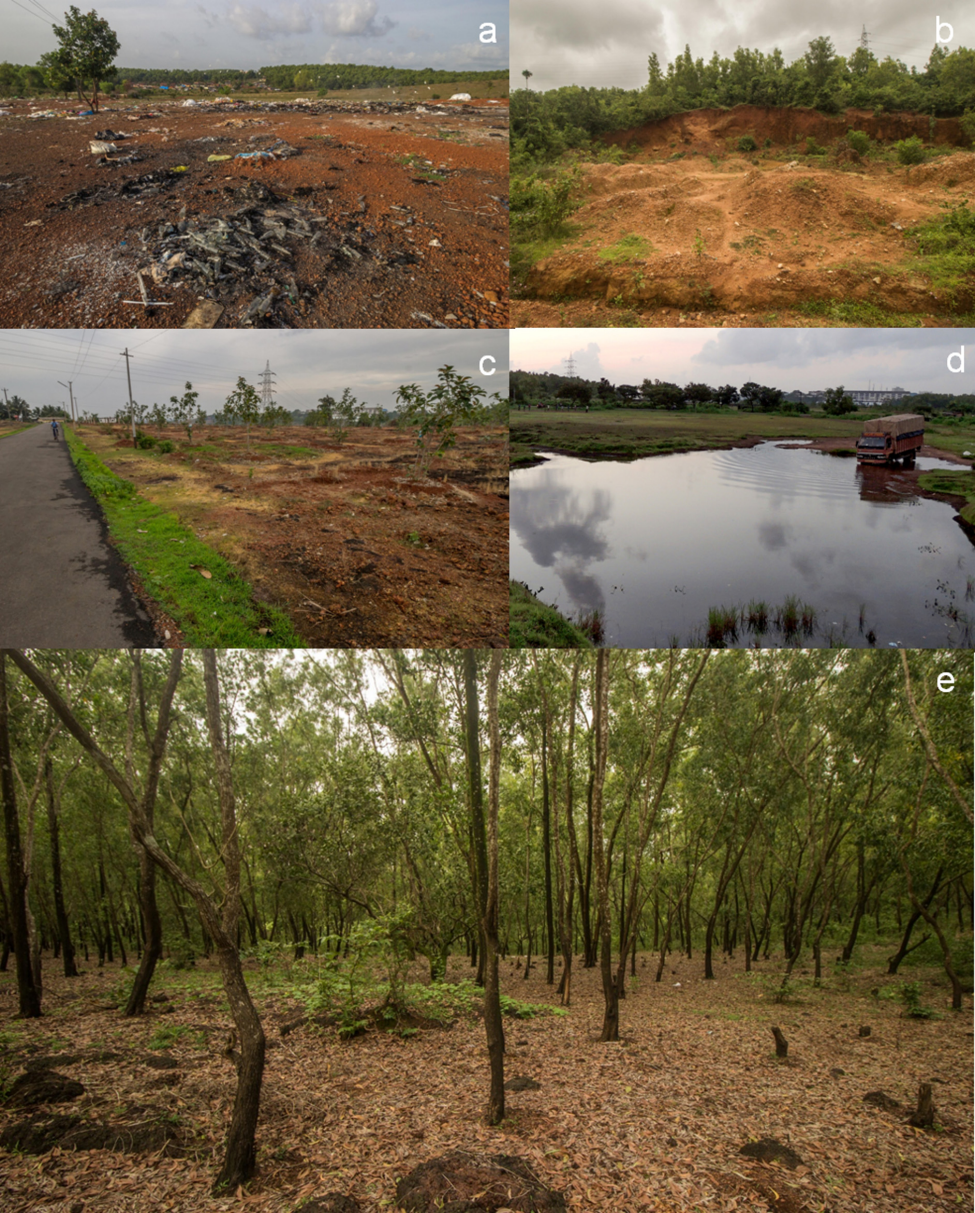
Depiction of land use change in Laterite habitats. (a) Garbage dumping. (b) Mining for Laterite. (c) Urbanization. (d) Washing vehicles. (e) Habitat converted to *Acacia* sp. plantation.

The localities where we observed the frog (Fig 1), are very few. They are dominated by shrubs, grasses and woody trees like S*trychnos nux*-*vomica*, *Careya arborea*, *Macaranga indica*, *Mangifera indica* and plantations of *Acacia* spp., *Gliricidia* spp. and *Syzygium* spp. The geographic range of *M*. *laterite* is small (146.13 km^2^) and warrants immediate conservation attention. We list this species as endangered (EN) under the IUCN Red List criteria B1ab(iii)(iv) where the estimated extent of occurrence is less than 500 km^2^, the habitat is severely fragmented, there is a continued decline of area, extent and/or quality of habitat and the species is present in less than five locations. Surveys in other regions along the West coast have not detected this species. Laterite habitats receive very little protection from any legislation and are sought after for developmental works and are heavily mined for construction material in form of bricks [36]. They are also subject to destructive anthropogenic activities like dumping of municipal solid wastes; fuel wood collection; conversion to plantations; encroachments; use of ephemeral pools for domestic ablutions and washing of vehicles (Fig 6). All these severely impact the already fragmented habitat and cause irreparable damages.

### Conservation Interventions

Laterite areas in India receive no protection and are considered as wastelands [38]. Given the threats these fragile habitats are facing, it is imperative to conserve them. The Wildlife Protection Act (1972) in conjunction with the National Wildlife Action Plan (2002–2016) provides opportunities for conserving such areas where they can be declared as ‘Conservation Reserves’ and/or ‘Community Reserves’. These interventions will prevent further degradation by halting land conversion. With Community Reserves, the local community members are empowered to be key stakeholders and be greatly involved in conservation action [40]. Further, other legislations like the Biological Diversity Act (2002), enable areas of biological importance to be protected as ‘Biological Heritage Areas’ and several regions have already been listed in this framework [41]. These opportunities need to be explored in order to conserve the fragile laterite habitats which are vital for *M*. *laterite*.

### Future Directions

The phylogenetic tree generated in this study provided low bootstrap support for almost all species except *M*. *sholigari*. The overall weak bootstrap support across species and clades is indicative of a spurious phylogenetic association because of missing species. Several attempts to understanding the phylogeny of species across taxa have encountered this issue. This arises either out of ‘Linnean shortfall’, where many un-described species exist or of ‘Wallacean shortfall’ where the geographic ranges are poorly understood due to lack of systematic sampling [42, 43]. Another added challenge here is the lack of relevant phylogenetic information of taxonomically valid species, known as the ‘Darwinian shortfall’ [44]. Therefore, it is imperative that systematic surveys are undertaken across Indian subcontinent, including Sri Lanka for a robust phylogeny and systematics of the genus *Microhyla*. These efforts would help in overcoming some of the limitations we currently face in understanding amphibian ecology and evolution.

With molecular tools becoming increasingly reliable and affordable; studies could shed light into the population dynamics of these small frogs found in isolated and severely fragmented landscapes. In context of laterite habitats, studies have estimated the early diversification period of Microhylidae to be at the late Cretaceous period and that of *Microhyla* to be in the lower Tertiary period; signifying that several lineages survived through the KT boundary [45]. Since *M*. *laterite* appears to be restricted to laterite rock formations along the West coast, further research on determining divergence times of *M*. *laterite* and testing for an association with laterite formations would enable a better understanding of biogeography, systematics and paleo-ecology. This will enable us to explore interesting evolutionary ecology questions in *Microhyla*.

## Supporting Information

S1 Appendix Details of abbreviations used for morphometric measurements. (DOCX)

S1 Audio Call record of *M*. *laterite* sp. nov. (WAV)

S2 Audio Call record of *M*. *sholigari*. (WAV)

S1 Table List of species and accession numbers used for phylogenetic analysis. List was based on Howlader, Nair (28).
(DOCX)

S2 Table Un-corrected pairwise genetic distances of *Microhyla* species used for analysis. *Uperodon variegatus* is used as an out-group. Genetic distances are in percentage.
(DOCX)

## Abbreviations

KSS: K S Seshadri
KVG: K V Gururaja
HP: H Priti
RS: Ramit Singal
IUCN: International Union for Conservation of Nature
BNHS: Bombay Natural History Society
