# Supplementary material for: Microhyla laterite sp. nov., A New Species of Microhyla Tschudi, 1838 (Amphibia: Anura: Microhylidae) from a Laterite Rock Formation in South West India
Source: PLoS One. 2016 Mar 9;11(3):e0149727. doi: 10.1371/journal.pone.0149727 (PMC4784882; doi:10.1371/journal.pone.0149727)
Supplement: S1 Appendix — (DOCX) [file pone.0149727.s001.docx]

**S1 Appendix. Details of abbreviations used for morphometric measurements.**

Snout–vent length (SVL); head width, at the angle of the jaws (HW); head length, from the rear of the mandible to the tip of the snout (HL); inter upper eyelid width, i.e. the shortest distance between the upper eyelids (IUE); maximum upper eyelid width (UEW); snout length, measured from the tip of the snout to the anterior orbital border of the eye (SL); eye length, i.e. the horizontal distance between the bony orbital borders of the eye (EL); distance from the rear of the mandible to the nostril (MN); distance from the rear of the mandible to the anterior orbital border of the eye (MFE); distance from the rear of the mandible to the posterior orbital border of the eye (MBE); distance between anterior corner of eyes, i.e. the shortest distance between the anterior orbital borders of the eyes (IFE); distance between posterior corner of eyes, i.e. the shortest distance between the posterior orbital borders of the eyes (IBE); forelimb length, measured from the elbow to the base of the outer palmar tubercle (FLL); hand length, measured from the base of the outer palmar tubercle to the tip of the third finger (HAL); thigh length (TL); shank length (ShL); tibia width, i.e. width of tibia at its widest region (TW); foot length, measured from the base of the inner metatarsal tubercle to the tip of the fourth toe (FOL); distance from the heel to the tip of the fourth toe (TFOL); internarial distance, i.e. least distance between the inner margins of nares (IN); nostril–snout distance, i.e. distance between middle of nostril and tip of snout (NS); eye to nostril distance, i.e. distance between anterior-most point of eye and middle of nostril (EN); length of inner metatarsal tubercle (IMT); distance from distal edge of metatarsal tubercle to maximum incurvature of web between fourth and fifth toe (MTFF); distance from distal edge of metatarsal tubercle to maximum incurvature of web between third and fourth toe (MTTF); distance from maximum incurvature of web between fourth and fifth toe to tip of fourth toe (FFTF); distance from maximum incurvature of web between third and fourth toe to tip of fourth toe (TFTF).
