# Supplementary material for: Microhyla laterite sp. nov., A New Species of Microhyla Tschudi, 1838 (Amphibia: Anura: Microhylidae) from a Laterite Rock Formation in South West India
Source: PLoS One. 2016 Mar 9;11(3):e0149727. doi: 10.1371/journal.pone.0149727 (PMC4784882; doi:10.1371/journal.pone.0149727)
Supplement: S1 Table — List was based on Howlader, Nair (28). (DOCX) [file pone.0149727.s004.docx]

**S1 Table. List of species and accession numbers used for phylogenetic analysis. List was based on Howlader, Nair (28).**

| **Sl. No** | | **Species** | | **Voucher** | | **16S** | | **12S** | |
| --- | --- | --- | --- | --- | --- | --- | --- | --- | --- |
| **1** | *M. achatina* | | MZBamp16401 | | AB598335 | | AB598311 | |  |
| **2** | *M. annectens* | | KUHE52438 | | AB634659 | | AB634601 | |  |
| **3** | *M. berdmorei* | | KUHE | | AB598338 | | AB598314 | |  |
| **4** | *M. butleri* | | KUHE33557 | | AB201189 | | AB201178 | |  |
| **5** | *M. fissipes* | | KUHE35165 | | AB201186 | | AB201175.1 | |  |
| **6** | *M. fowleri* | | KUHE21992 | | AB634667 | | AB634609 | |  |
| **7** | *M. heymonsi* | | KUHEK1845 | | AB201179 | | ­AB201179.1 | |  |
| **8** | *M. malang* | | KUHE42597 | | AB598322 | | AB598298 | |  |
| **9** | *M. mantheyi* | | KUHE52556 | | AB598334 | | AB598310 | |  |
| **10** | *M. marmorata* | | KUHE32455 | | AB611955 | | AB634610 | |  |
| **11** | *M. mixtura* | | CIB | | AB634669 | | AB634611 | |  |
| **12** | *M. nilphamariensis* | | MZH-2360-66 | | KP072787 | | ­NA | |  |
| **13** | *M. mukhlesuri* | | IABHU3880 | | AB543609.1 | | NA | |  |
| **14** | *M. okinavensis* | | KUHE12840 | | AB201184.1 | | AB201173.1 | |  |
| **15** | *M. ornata* | | ZSI-A9119 | | AB201188 | | AB201177 | |  |
| **16** | *M. palmipes* | | MZBAAmp16323 | | AB634671 | | AB634613 | |  |
| **17** | *M. perparva* | | KUHE:53675 | | AB634673 | | AB634615 | |  |
| **18** | *M. petrigena* | | KUHE53743 | | AB634675 | | AB634617 | |  |
| **19** | *M. pulchra* | | KUHE35119 | | AB201191 | | AB201180 | |  |
| **20** | *M. rubra* | | ­NA | | AB201192 | | AB201181.1 | |  |
| **21** | *M. superciliaris* | | KUHE52558 | | AB634682 | | AB634624 | |  |
| **22** | *M. sholigari_1* | | ATREE_MISH_1 | | KT600674 | | KT600667 | |  |
| **23** | *M. sholigari_2* | | ATREE_MISH_2 | | KT600675 | | KT600668 | |  |
| **24** | *M. sholigari_3* | | ATREE_MISH_3 | | KT600676 | | KT600669 | |  |
| **25** | *M. sholigari_4* | | ATREE_MISH_4 | | KT600672 | | KT600665 | |  |
| **26** | *M. sholigari_5* | | ATREE_MISH_5 | | KT600673 | | KT600666 | |  |
| **27** | *M. laterite* sp. nov. | | BNHS 5965 | | KT600670 | | KT600663 | |  |
| **28** | *M. laterite* sp. nov. | | BNHS 5967 | | KT600671 | | KT600664 | |  |
| **29** | *Uperodon variegatus* | | ­NA | | GU136114 | | GU136098 | |  |
