# Supplementary material for: Microhyla laterite sp. nov., A New Species of Microhyla Tschudi, 1838 (Amphibia: Anura: Microhylidae) from a Laterite Rock Formation in South West India
Source: PLoS One. 2016 Mar 9;11(3):e0149727. doi: 10.1371/journal.pone.0149727 (PMC4784882; doi:10.1371/journal.pone.0149727)
Supplement: S2 Table — Uperodon variegatus is used as an out-group. Genetic distances are in percentage. (DOCX) [file pone.0149727.s005.docx]

|  | *Uperodon variegatus* | *M. achatina* | *M. annectens* | *M. berdmorei* | *M. butleri* | *M. fissipes* | *M. fowleri* | *M. heymonsi* | *M. malang* | *M. mantheyi* | *M. marmorata* | *M. mixtura* | *M. nilphamariensis* | *M. mukhlesuri* | *M. okinavensis* | *M. ornata* | *M. palmipes* | *M. perparva* | *M. petrigena* | *M. pulchra* | *M. rubra* | *M. superciliaris* | *M. laterite_1 sp. nov.* | *M. laterite_2 sp. nov.* | *M. sholigari_1* | *M. sholigari_2* | *M. sholigari_3* | *M. sholigari_4* |
| --- | --- | --- | --- | --- | --- | --- | --- | --- | --- | --- | --- | --- | --- | --- | --- | --- | --- | --- | --- | --- | --- | --- | --- | --- | --- | --- | --- | --- |
| *M. achatina* | 14.57 |  |  |  |  |  |  |  |  |  |  |  |  |  |  |  |  |  |  |  |  |  |  |  |  |  |  |  |
| *M. annectens* | 11.59 | 11.78 |  |  |  |  |  |  |  |  |  |  |  |  |  |  |  |  |  |  |  |  |  |  |  |  |  |  |
| *M. berdmorei* | 15.62 | 7.49 | 9.13 |  |  |  |  |  |  |  |  |  |  |  |  |  |  |  |  |  |  |  |  |  |  |  |  |  |
| *M. butleri* | 14.59 | 11.31 | 10.32 | 10.58 |  |  |  |  |  |  |  |  |  |  |  |  |  |  |  |  |  |  |  |  |  |  |  |  |
| *M. fissipes* | 15.11 | 7.28 | 11.30 | 8.91 | 10.33 |  |  |  |  |  |  |  |  |  |  |  |  |  |  |  |  |  |  |  |  |  |  |  |
| *M. fowleri* | 15.61 | 7.50 | 10.09 | 2.13 | 10.82 | 9.62 |  |  |  |  |  |  |  |  |  |  |  |  |  |  |  |  |  |  |  |  |  |  |
| *M. heymonsi* | 15.37 | 7.76 | 10.81 | 9.38 | 10.81 | 5.89 | 9.15 |  |  |  |  |  |  |  |  |  |  |  |  |  |  |  |  |  |  |  |  |  |
| *M. malang* | 15.37 | 7.09 | 12.29 | 10.35 | 10.33 | 7.04 | 9.87 | 5.44 |  |  |  |  |  |  |  |  |  |  |  |  |  |  |  |  |  |  |  |  |
| *M. mantheyi* | 14.84 | 5.92 | 12.03 | 9.62 | 11.07 | 7.28 | 9.14 | 7.73 | 6.37 |  |  |  |  |  |  |  |  |  |  |  |  |  |  |  |  |  |  |  |
| *M. marmorata* | 11.66 | 10.82 | 6.12 | 10.81 | 9.37 | 9.62 | 11.05 | 10.09 | 9.85 | 11.07 |  |  |  |  |  |  |  |  |  |  |  |  |  |  |  |  |  |  |
| *M. mixtura* | 16.15 | 6.34 | 11.29 | 8.90 | 9.37 | 4.10 | 9.14 | 5.66 | 6.80 | 6.14 | 9.13 |  |  |  |  |  |  |  |  |  |  |  |  |  |  |  |  |  |
| *M. nilphamariensis* | 14.40 | 9.14 | 11.30 | 8.90 | 9.14 | 9.17 | 9.13 | 10.10 | 10.10 | 9.86 | 10.10 | 8.21 |  |  |  |  |  |  |  |  |  |  |  |  |  |  |  |  |
| *M. mukhlesuri* | 15.12 | 6.82 | 11.55 | 9.40 | 9.85 | 1.92 | 10.11 | 5.67 | 6.82 | 7.28 | 9.63 | 4.32 | 8.69 |  |  |  |  |  |  |  |  |  |  |  |  |  |  |  |
| *M. okinavensis* | 15.60 | 7.04 | 13.03 | 9.38 | 11.81 | 5.69 | 9.14 | 7.06 | 7.97 | 7.29 | 11.07 | 3.23 | 9.88 | 6.40 |  |  |  |  |  |  |  |  |  |  |  |  |  |  |
| *M. ornata* | 14.43 | 10.57 | 11.56 | 9.84 | 11.07 | 9.62 | 10.57 | 10.12 | 11.30 | 12.04 | 9.88 | 9.85 | 4.99 | 8.67 | 10.34 |  |  |  |  |  |  |  |  |  |  |  |  |  |
| *M. palmipes* | 14.10 | 11.57 | 10.36 | 11.56 | 9.89 | 7.96 | 11.55 | 9.14 | 11.07 | 11.81 | 9.40 | 9.15 | 8.71 | 8.67 | 10.36 | 8.47 |  |  |  |  |  |  |  |  |  |  |  |  |
| *M. perparva* | 11.84 | 13.29 | 8.01 | 12.55 | 10.32 | 11.31 | 12.55 | 10.58 | 12.04 | 13.04 | 6.57 | 11.06 | 12.30 | 10.58 | 12.30 | 11.56 | 9.63 |  |  |  |  |  |  |  |  |  |  |  |
| *M. petrigena* | 11.07 | 12.30 | 7.73 | 10.85 | 11.30 | 10.62 | 11.32 | 10.35 | 12.06 | 12.56 | 7.27 | 10.82 | 11.35 | 10.13 | 12.59 | 11.10 | 9.63 | 5.46 |  |  |  |  |  |  |  |  |  |  |
| *M. pulchra* | 15.12 | 7.99 | 11.55 | 7.03 | 9.85 | 8.23 | 7.49 | 7.97 | 9.88 | 10.35 | 10.58 | 7.74 | 8.45 | 8.23 | 8.44 | 8.91 | 9.87 | 12.06 | 11.82 |  |  |  |  |  |  |  |  |  |
| *M. rubra* | 15.37 | 9.63 | 12.03 | 10.10 | 9.39 | 8.69 | 9.85 | 9.64 | 9.62 | 9.62 | 9.61 | 9.40 | 6.14 | 8.46 | 9.66 | 8.23 | 9.90 | 12.53 | 10.83 | 8.92 |  |  |  |  |  |  |  |  |
| *M. superciliaris* | 14.11 | 11.31 | 11.30 | 10.33 | 7.26 | 9.14 | 10.57 | 8.90 | 11.06 | 10.59 | 10.60 | 9.39 | 9.15 | 8.43 | 11.37 | 9.65 | 8.45 | 11.06 | 9.85 | 10.58 | 8.44 |  |  |  |  |  |  |  |
| *M. laterite_1 sp. nov.* | 14.45 | 12.82 | 14.11 | 12.06 | 12.12 | 11.56 | 12.81 | 12.30 | 12.54 | 11.80 | 11.38 | 11.81 | 11.81 | 11.56 | 13.31 | 12.12 | 12.36 | 13.83 | 14.14 | 12.85 | 11.31 | 10.38 |  |  |  |  |  |  |
| *M. laterite_2 sp. nov.* | 14.45 | 12.82 | 14.11 | 12.06 | 12.12 | 11.56 | 12.81 | 12.30 | 12.54 | 11.80 | 11.38 | 11.81 | 11.81 | 11.56 | 13.31 | 12.12 | 12.36 | 13.83 | 14.14 | 12.85 | 11.31 | 10.38 | 0.00 |  |  |  |  |  |
| *M. sholigari_1* | 15.22 | 13.09 | 13.09 | 11.32 | 11.63 | 11.56 | 10.59 | 12.06 | 12.81 | 12.06 | 12.12 | 11.57 | 10.34 | 11.81 | 13.85 | 11.09 | 11.60 | 14.61 | 13.09 | 11.83 | 9.85 | 10.38 | 5.03 | 5.03 |  |  |  |  |
| *M. sholigari_2* | 14.95 | 12.58 | 12.58 | 11.07 | 11.63 | 11.31 | 10.34 | 11.81 | 12.81 | 11.56 | 12.12 | 11.32 | 10.58 | 11.56 | 13.59 | 11.09 | 11.60 | 14.61 | 12.84 | 12.08 | 9.61 | 10.13 | 5.03 | 5.03 | 0.42 |  |  |  |
| *M. sholigari_3* | 15.22 | 12.84 | 12.84 | 11.07 | 11.38 | 11.31 | 10.34 | 11.81 | 12.55 | 11.81 | 11.86 | 11.57 | 10.34 | 11.56 | 13.59 | 10.84 | 11.35 | 14.35 | 12.84 | 11.83 | 9.61 | 10.13 | 4.80 | 4.80 | 0.21 | 0.21 |  |  |
| *M. sholigari_4* | 15.22 | 12.84 | 12.84 | 11.07 | 11.38 | 11.31 | 10.34 | 11.81 | 12.55 | 11.81 | 11.86 | 11.57 | 10.34 | 11.56 | 13.59 | 10.84 | 11.35 | 14.35 | 12.84 | 11.83 | 9.61 | 10.13 | 4.80 | 4.80 | 0.21 | 0.21 | 0.00 |  |
| *M. sholigari_5* | 15.22 | 12.84 | 12.84 | 11.07 | 11.38 | 11.31 | 10.34 | 11.81 | 12.55 | 11.81 | 11.86 | 11.57 | 10.34 | 11.56 | 13.59 | 10.84 | 11.35 | 14.35 | 12.84 | 11.83 | 9.61 | 10.13 | 4.80 | 4.80 | 0.21 | 0.21 | 0.00 | 0.00 |

**S2 Table. Un-corrected pairwise genetic distances of *Microhyla* species used for analysis. *Uperodon variegatus* is used as an out-group. Genetic distances are in percentage.**
